# Association of Dietary Patterns, *C*-Reactive Protein, and Risk of Obesity Among Children Aged 9–17 Years in Guangzhou, China: A Cross-Sectional Mediation Study

**DOI:** 10.3390/nu16223835

**Published:** 2024-11-08

**Authors:** Zheng Su, Chunzi Zeng, Jie Huang, Shiyun Luo, Jiaying Guo, Jinhan Fu, Weiwei Zhang, Zhoubin Zhang, Bo Zhang, Yan Li

**Affiliations:** 1 School of Public Health, Southern Medical University, Guangzhou 510515, China; sue1995@smu.edu.cn (Z.S.); zhangbo2018@smu.edu.cn (B.Z.); 2 Department of Foodborne Diseases and Food Safety Risk Surveillance, Guangzhou Center for Disease Control and Prevention, Guangzhou 510440, China; gzcdc_zengcz@gz.gov.cn (C.Z.); huangjie1026@126.com (J.H.); luoshy25@mail3.sysu.edu.cn (S.L.); gzcdczhangww@foxmail.com (W.Z.); zhang_zhoubin@163.com (Z.Z.); 3 School of Public Health, Sun Yat-sen University, Guangzhou 510080, China; guojy78@mail2.sysu.edu.cn (J.G.); fujh6@mail2.sysu.edu.cn (J.F.)

**Keywords:** dietary pattern, *C*-reactive protein, obesity, mediation analysis, children, China

## Abstract

Background: Childhood obesity is a major public health challenge in the 21st century, and diet is one of the key modifiable factors in its prevention. This study examined the link between dietary patterns of children and general and central obesity, including the role of *C*-reactive protein (CRP). Methods: This study enrolled 2413 children aged 9–17. Anthropometric measurements, CRP levels, and dietary data were collected. Factor analysis identified dietary patterns, and logistic regression examined the association between CRP levels and childhood obesity. Multiple linear regression determined the correlation between dietary patterns and CRP. Mediation analysis assessed the role of CRP in the link between dietary patterns and childhood obesity. Results: Three dietary patterns were identified. The rice and meat pattern was significantly correlated to the risk of childhood obesity (OR = 1.166, 95%CI: 1.000, 1.359 for general obesity; OR = 1.215, 95%CI: 1.071, 1.377 for central obesity). CRP was positively correlated with childhood obesity risk (OR = 2.301, 95%CI: 1.776, 2.982 for general obesity; OR = 2.165, 95%CI: 1.738, 2.697 for central obesity). The fruit and vegetable pattern was inversely related to CRP (β= −0.059, 95%CI: −0.081, −0.036), while the snack pattern was positively correlated (β= 0.043, 95%CI: 0.020, 0.065). CRP had a suppressive effect on the association between the fruit and vegetable pattern and snack pattern with childhood obesity. Conclusions: This study revealed the rice and meat pattern as a risk factor for childhood obesity, and cross-sectional evidence linked the fruit and vegetable pattern and snack pattern to childhood obesity risk, mediated by CRP.

## 1. Introduction

Obesity is a chronic disorder closely linked to type 2 diabetes, hypertension, and cardiovascular disease [1]. Studies have reported that the state of obesity tends to continue from childhood to adulthood [2]. A follow-up study of Chinese children showed that childhood central obesity significantly increases the risk of adult central obesity [3]. Obesity in childhood and adolescence is associated with an increased risk of type 2 diabetes and cardiovascular disease in adulthood [4]. Over the last 40 years, there has been a significant increase in the prevalence of obesity among children globally, with the rate rising from 0.7% to 5.6% for girls and from 0.9% to 7.8% for boys [5]. The prevalence of obesity in Chinese children increased from 0.1% in 1985 to 9.6% in 2019 [6].

Diet is a pivotal modifiable factor in the treatment of obesity [7]. Compared to focusing on single nutrients or specific foods, dietary patterns offer a more holistic perspective of food combinations, their synergies and antagonisms, and may establish dietary recommendations that are more acceptable to the public [8]. Due to the different food cultures of different regions, residents of the Jiangnan region of China are more inclined to accept the Jiangnan dietary pattern that is as healthy as the Eastern Mediterranean dietary pattern [9]. The United States Heart Association’s 2021 Dietary Guidelines highlight that dietary patterns have more health implications than a single food or nutrient [10]. Moreover, dietary patterns are becoming the core concept of multinational dietary guidelines [11]. A systematic review of intervention and observational studies showed that the Mediterranean diet based on fruits and vegetables, fish, whole grains, legumes, and olive oil helped children maintain a healthy weight [12]. A meta-analysis incorporating several randomized trials pointed out that over a 6-month follow-up period, the Dietary Approaches to Stop Hypertension (DASH) diet was associated with weight loss in adults [13].

The pathophysiology of obesity is complex and not fully understood [14]. Obesity is a systemic chronic low-grade inflammatory state induced by a variety of inflammatory factors, which are closely associated with specific dietary patterns and major food groups. Evidence from a systematic review suggests that a high-energy-density, high-fat, and low-fiber diet pattern in childhood increases the risk of obesity later in life [15]. Pro-inflammatory diets are also regarded as one of the key pathways to obesity, where such diets are known to elevate inflammation levels within the body, potentially altering metabolism, leading to fat accumulation, and consequently increasing the risk of obesity [16]. Diet influences CRP, a significant inflammatory marker, which is correlated with the risk of obesity [17].

The findings of studies on dietary patterns and obesity are conflicting, implying that other factors may influence the dietary effect on obesity [18]. There is a scarcity of research on how CRP affects the relationship between dietary patterns and obesity in children. Central obesity, which is more common in children, has a strong influence on cardiometabolic disorders such as hypertension, insulin resistance, and dyslipidemia. However, it has been studied less extensively than general obesity [14]. Furthermore, there have been fewer studies on the relationship between dietary patterns and central obesity in children than general obesity.

The aim of this study was to investigate the correlation between the dietary patterns of children and both general and central obesity, as well as to explore the link between dietary patterns and CRP and the function of CRP in the association between dietary patterns and childhood obesity.

## 2. Materials and Methods

### 2.1. Participants

This cross-sectional investigation was conducted between April 2022 and June 2023. Participants were recruited using a multi-stage stratified cluster random sampling method: (1) Five primary schools, five junior high schools, and two senior high schools were randomly sampled from rural areas in Guangzhou. (2) Five grades were selected using stratified sampling, including two grades from each primary school, two grades from each junior high school, and a single grade from each senior high school. (3) Two to three classes of students were randomly sampled for each grade.

**Figure 1:**
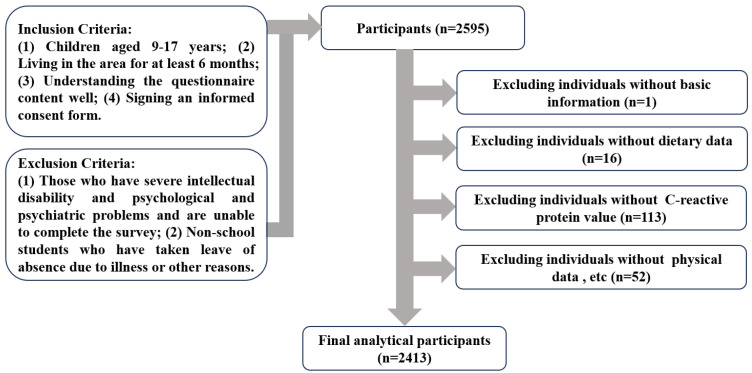
Flowchart of the subject selection process.

The sample size was determined using the following formula: N = deff $\frac{u_{\alpha /2}^{2}P(1-P)}{\delta^{2}}$ [19]. The definitions and numerical values of the parameters are described below: the confidence level was set to 95% (two-tailed), with the corresponding u_(α/2) = 1.96. In 2018, the prevalence rate of general obesity in children in Guangzhou was 6.2% [20]. The probability *p* was set at 6%, the design efficiency value (deff) was 1.4, the relative error (r) was 20%, and δ = 20% × 6%. Using these parameter values, the calculated N value was 2106. The actual sample size was increased by 10% to account for the rejection rate and invalid questionnaires, resulting in a final N = 2106 × 110% = 2317. A total of 2413 subjects were included in the analysis (Figure 1). The present study adhered to the Declaration of Helsinki and received approval from the Ethics Committee of Guangzhou Center for Disease Control and Prevention (ethics number GZCDC-ECHR-2021P0019 and GZCDC-ECHR-2022P0038). Informed consent forms were signed by all participants and their legal guardians.

### 2.2. Survey Content

Surveyors who underwent uniform training conducted face-to-face surveys with children. The surveys included questionnaires, physical measurements, and laboratory tests.

(1) Questionnaires: Basic information included gender, age, region, and parental educational level. The survey on lifestyle factors was derived from the questionnaire of the “Monitoring and Intervention Work Plan for Common Diseases and Health-Influencing Factors of Chinese Students”, which was widely used [21]. The indicators and definitions in the survey form are as follows: (1) Tried smoking refers to whether an individual has ever smoked cigarettes, including taking just one or two puffs. (2) Alcohol consumption refers to whether an individual has ever consumed a full cup of alcohol, where the volume of a full cup is equivalent to one can of beer, one small glass of liquor, or one glass of wine or yellow wine. (3) Moderate to vigorous physical activity is measured by the question, “On how many days per week do you engage in at least 60 min of moderate to vigorous physical activity?” (Moderate to vigorous physical activity is defined as exercise that causes shortness of breath or an increased heart rate, such as running, playing basketball, soccer, swimming, aerobics, or lifting heavy objects). Based on the “Dietary Guidelines for Chinese School-Aged Children (2022)”, engaging in such activity for less than three days a week is considered below the recommended standard [22]. (4) Screen time is measured by the question, “How many hours per day do you spend on screen time, which includes the use of mobile phones, computers, tablets, TVs, and similar electronic devices?” According to the “Dietary Guidelines for Chinese School-Aged Children (2022)”, a daily screen time of two hours or more is considered excessive [22]. (5) Sleep time is measured by the questions, “What time do you wake up? What time do you go to bed?” In accordance with the Chinese national standard “Health requirements of daily learning time for secondary and elementary school students (GB/T 17223-2012)”, sleep times of less than 10 h for primary school students, 9 h for junior high school students, and 8 h for senior high school students are considered insufficient [23]. (6) Bedtime is measured by the question, “What time do you go to bed?” Going to bed after 22:30 is considered a late bedtime.
  A semi-quantitative food frequency questionnaire (FFQ) was used to assess the dietary intake of children over the previous month, including both the frequency and quantity of food consumed. Participants were provided with photos and models of food to assist them in determining portion sizes. The questionnaire was based on the food frequency survey established by the China National Center for Chronic Noncommunicable Disease and Nutrition Surveillance [24] and was amended by a team of experts to reflect the dietary habits of Guangzhou children. The FFQ employed in this study included 66 food items across 20 major categories, based on the Chinese Food Composition List (6th Edition) [25].
(2) Physical Measurements: The height was measured using a metallic column stature meter with a 0.1 cm precision. Weight was measured using an electronic scale with a precision of 0.1 kg. The waist circumference was assessed using a glass fiber tape measure with a 0.1 cm precision. Physical measurements were conducted in compliance with the technical standards for the physical examination of students [26]. The body mass index (BMI) was calculated by dividing the weight (kg) by the square of the height (m). Based on the Chinese Health Industry Standards Screening for Overweight and Obesity among School-Aged Children and Adolescents (WS/T586—2018) [27], age- and gender-specific BMI standards were utilized to determine general obesity, and based on the High Waist Circumference Screening Threshold Among Children and Adolescents Aged 7–18 years (WS/T611—2018) [28], age- and gender-specific waist circumference standards were used to determine central obesity.
(3) Laboratory Testing: Venous blood was collected early in the morning on the day of the survey. The supernatant serum was collected and stored at −80 degrees Celsius. The serum CRP concentrations were measured using an immunoturbidimetric assay in a BS2000M fully automatic biochemical analyzer from Mindray Corporation, Shenzhen, China.

### 2.3. Dietary Pattern Establishment

Dietary patterns were established using exploratory factor analysis, by categorizing 66 food items into 20 food groups (Supplementary Materials Table S1). A statistical test was performed on the correlation matrix among the 20 food groups. The Kaiser–Meyer–Olkin test result was >0.8, and Bartlett’s test of sphericity was significant (*p* < 0.001), indicating that factor analysis could be conducted. Principal component analysis was used to extract common factors which were then rotated using varimax rotation. The number of dietary patterns was determined using an eigenvalue of >1, a scree plot, and expert knowledge. In this study, factors with absolute factor loadings greater than 0.3 were considered integral components of the dietary patterns. The standardized factor scores for each pattern indicated the extent to which the participants adhered to the specific dietary trend.

### 2.4. Lifestyle Model Establishment

A lifestyle model was established using latent class analysis. The latent class analysis operates on parameter estimation and uses individual response patterns to observable indicators, which are represented by variable joint probabilities [29]. The critical parameters in this analysis were the latent class probabilities, which account for the proportion of individuals in each category, and the conditional probabilities, indicative of the probability of individuals within a latent class responding positively to the observed indicators [30]. The lifestyle model utilized in this study consisted of six variables, including tried smoking, alcohol consumption, moderate to high physical activity, screen time, sleep time, and bedtime. The six measurable indicators were categorized into binary variables according to relevant guidelines and standards, and then included in the latent class model analysis. Detailed assignment information can be found in Supplementary Materials Table S2.

### 2.5. Statistical Analysis

A unified coding system was implemented for the questionnaires and a database was established using EpiData version 3.1. Data entry was performed by two individuals. Microsoft Excel 2019 was used to ensure comprehensive and logically consistent data. Descriptive data for baseline characteristics are represented by the mean (standard deviation) for continuous variables or the median (interquartile range), and categorical variables are presented as frequencies (percentages). Intergroup differences were compared using independent sample *t*-tests, Mann–Whitney U tests, or chi-square tests. Factor analysis was used to construct the dietary patterns. The lifestyle model was established using latent class analysis. Because the CRP data were skewed, a logarithmic transformation was performed before further analysis.

Mediation analysis was performed using Wen’s approach to investigate how CRP affects the relationship between dietary patterns (independent variable) and general and central obesity (dependent variables) [31]. The process involved several steps: (1) Logistic regression analysis was used to test the regression coefficient c for the total effect of each dietary pattern on general and central obesity. If significant, the mediation effect was pursued; if not, the suppression effect was pursued. Regardless of the significance, subsequent tests were conducted. (2) In the mediation model, the regression coefficient a of each dietary pattern with CRP was tested using multiple linear regression analysis, followed by the regression coefficient b of CRP with general and central obesity using logistic regression analysis. If both a and b were significant, the indirect effect was considered significant, and we moved to step four; if at least one was not significant, we proceeded to step three. (3) We directly tested H0: ab = 0 using the bootstrap method (times = 5000). If the indirect effect was significant, the analysis proceeded; if not, the analysis was stopped. (4) The regression coefficient c’ was tested to determine the direct effect of each dietary pattern on general and central obesity. If not significant, it indicated only an indirect effect; if the direct effect was significant, we proceeded to step five. (5) The signs of ab and c’ were compared to determine the mediation effect. If they had the same sign, it was a partial mediation effect; if they had different signs, it was a suppression effect. To control the impact of confounding factors, adjustments were made for gender, age, lifestyle, breakfast frequency, and the nature of the school attended.

The statistical analyses were conducted using IBM SPSS 27.0. The latent class model for lifestyle was constructed using Mplus 8.3. The mediation model between dietary patterns and general and central obesity was constructed using the mediation package in R language version 4.3.3. All statistical tests were two-tailed and differences were considered statistically significant at *p* < 0.05.

## 3. Results

### 3.1. Participant Characteristics

A total of 2413 children with complete datasets were enrolled in this study, comprising 46.2% girls (n = 1116). The age distribution ranged from 9 to 17 years, with 13.3 years being the median. The prevalence rates of general and central obesity were 6.8% and 10.2%, respectively (Table 1). Children with general obesity were characterized by being boys, having a lower frequency of breakfast consumption, having a mother with a higher level of education, attending private schools, and higher body mass index and waist circumference levels (*p* < 0.05), as compared to ones without general obesity. Compared to children without central obesity, those with central obesity were characterized by being boys, having a lower frequency of breakfast consumption, attending private schools, and higher body mass index and waist circumference levels (*p* < 0.05).

### 3.2. Lifestyle Model

This study constructed latent class models ranging from M2 (two latent classes) to M5 (five latent classes). The model included six measured lifestyle behaviors: tried smoking, alcohol consumption, moderate-to-high physical activity, screen time, sleep time, and bedtime. Model fit was assessed using indices such as the Akaike Information Criterion (AIC), Bayesian Information Criterion (BIC), Adjusted Bayesian Information Criterion (ABIC), Lo–Mendell–Rubin Likelihood Ratio Test (LMR), and Bootstrap Likelihood Ratio Test (BLRT). The findings indicated that for the M4 model, the *p*-value corresponding to the LMR was >0.05, indicating that the M3 model had a better fit than the M4 model. Given the simplicity of the model and the interpretability of the outcomes, the M3 model with three subclasses was selected (Supplementary Materials Table S3).

**Figure 2:**
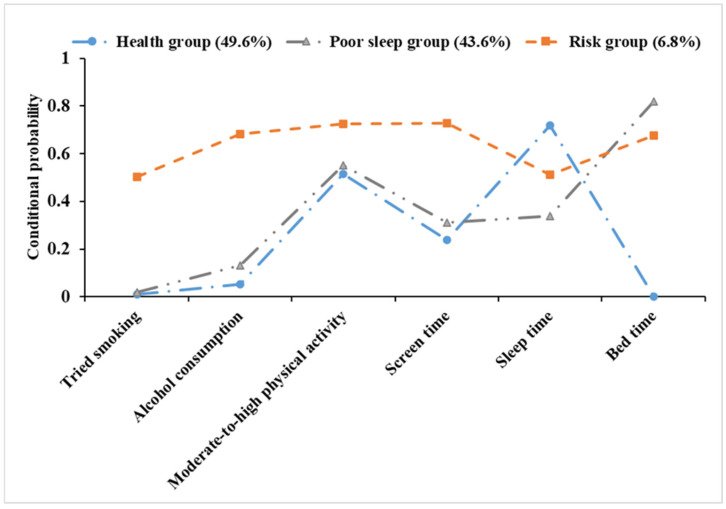
Conditional probability distribution of three latent classes.

The lifestyle model constructed in this study included three classes (Figure 2). Model 1 comprised 1196 individuals, accounting for 49.6% of the sample. This group was known as the “health group” since it had lower conditional probabilities for tried smoking, alcohol consumption, lengthy screen time, and late bedtime, but a higher conditional probability for adequate sleep. Model 2 comprised 1053 individuals, accounting for 43.6% of the sample. This group was known as the “poor sleep group” as it had a higher conditional probability for insufficient sleep and late bedtime. Model 3 included 164 individuals, accounting for 6.8% of the sample. This group was named the “risk group” as it exhibited higher conditional probabilities for tried smoking, alcohol consumption, long screen time, insufficient sleep, and late bedtime.

### 3.3. Dietary Patterns

**Figure 3:**
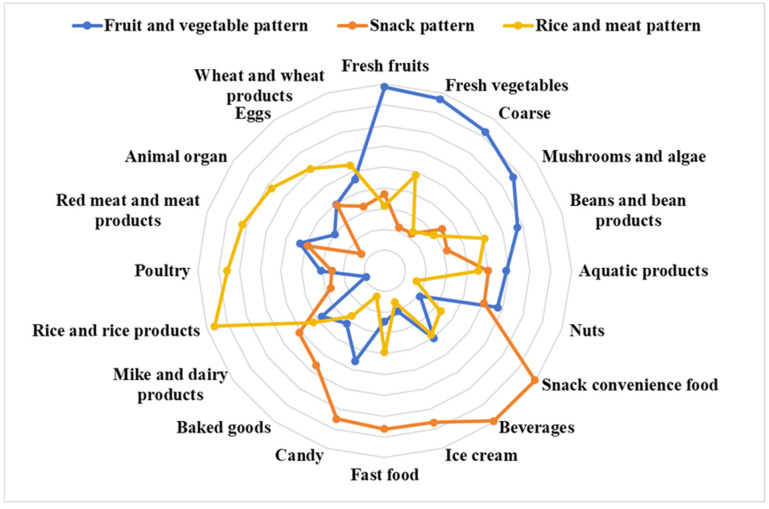
Radar charts of different dietary patterns obtained from factor analysis.

Three dietary patterns were identified using principal component analysis (Supplementary Materials Table S4 and Figure 3). Factor analysis distinguished three major dietary patterns among the 20 food groups, accounting for 12.4% (fruit and vegetable pattern), 12.4% (snack pattern), and 9.7% (rice and meat pattern) of the variance, which together explained 34.5% of the total variance. The matrix representing the factor loadings for the food groups was obtained by implementing the varimax orthogonal rotation to the factor component matrix (Supplementary Materials Table S4). The fruit and vegetable pattern was typified by fresh fruits, vegetables, whole grains, and fungi and algae; the snack pattern was typified by convenience foods, beverages, ice cream, fast food, and candy; and the rice and meat pattern was typified by rice, poultry, red meat, and their products (Figure 3).

### 3.4. Analysis of Dietary Patterns, CRP, and Obesity

The logistic regression analysis revealed that after adjusting for gender, age, lifestyle, frequency of breakfast consumption, and type of school attended, the higher the inclination towards a rice and meat dietary pattern, the higher the risk of general and central obesity amongst children (OR = 1.166, 95%CI: 1.000, 1.359 for general obesity; OR = 1.215, 95%CI: 1.071, 1.377 for central obesity). Furthermore, CRP showed a positive correlation with both general and central obesity (OR = 2.301, 95%CI: 1.776, 2.982 for general obesity; OR = 2.165, 95%CI: 1.738, 2.697 for central obesity) (Table 2).

### 3.5. Analysis of Dietary Patterns and CRP

The multiple linear regression analysis revealed that after adjusting for gender, age, lifestyle, frequency of breakfast consumption, and type of school attended, the propensity for fruit and vegetable pattern was negatively correlated with CRP (β = −0.059, 95%CI: −0.081, −0.036), while the propensity for the snack pattern was positively correlated (β = 0.043, 95%CI: 0.020, 0.065) (Table 3).

### 3.6. Mediation Analysis

The propensity for fruit and vegetable pattern was not substantially linked to the total effect on general and central obesity. The fruit and vegetable pattern showed an indirect negative effect on general and central obesity through the CRP pathway, demonstrating that CRP suppresses the relationship between the fruit and vegetable pattern and general and central obesity (Table 4). The preference for a snack pattern was not significantly related to the total effect on general and central obesity. The snack pattern showed an indirect positive effect on general and central obesity through the CRP pathway, with the indirect effect being in the opposite direction to the direct effect, implying that CRP plays a suppressive role between the snack pattern and general and central obesity (Table 5).

## 4. Discussion

In this study, the prevalence rates of general and central obesity among children were 6.8% and 10.2%, respectively. The obese group was characterized by the presence of boy students, infrequent breakfast consumption, and attendance at private schools. Three dietary patterns were identified including the fruit and vegetable pattern, the snack pattern, and the rice and meat pattern. After adjusting for confounding factors, the rice and meat pattern was significantly related to an elevated risk of general and central obesity in children. CRP level was positively correlated with an increased risk of both types of obesity. Conversely, the fruit and vegetable pattern negatively correlated with CRP levels, while the snack pattern was positively correlated. The mediation analysis revealed that CRP significantly mediates the association between dietary patterns and childhood obesity, with a suppressor effect reported in both the fruit and vegetable and snack pattern.

Over the past few decades, China’s rapid economic and social growth has resulted in significant changes in the energy intake and expenditure behavior of children [32]. Our findings showed that boys had higher rates of general and central obesity than girls, which is consistent with other Chinese studies [14]. Surveys have reported that girls have higher obesity rates than boys in most sub-Saharan African, Oceanian, and some middle-income nations; however, boys have been found to have higher obesity rates than girls in all high-income countries and East and Southeast Asian countries. The observed discrepancy may be attributed to cultural differences, parenting practices, and societal norms regarding the body sizes of boys and girls [33]. Breakfast is regarded as the most important meal of the day since it provides nutrients after an overnight fast. Studies have linked breakfast consumption to weight control, cardiovascular and metabolic risk factors, and cognitive performance [34,35,36]. Research indicates that skipping breakfast or infrequent breakfast may increase the risk of obesity in children, consistent with the findings of our study [37]. Moreover, skipping breakfast has also been associated with an impaired postprandial insulin response, potentially increasing the risk of obesity by influencing insulin secretion and blood sugar regulation [38].

In our study, the rice and meat pattern was characterized by rice, poultry, red meat, and meat products. This pattern represents the nutritional transition that China has undergone in recent decades, from food scarcity to diets high in fat and refined carbohydrates [39]. Our findings revealed that the rice and meat pattern positively correlated with childhood obesity. Cross-sectional studies have also linked increased central obesity risk among children to the high intake of refined carbohydrates [40]. Evidence from interventions involving overweight or obese adults suggests that reducing carbohydrate intake can effectively contribute to weight loss [41]. Despite ongoing debates, the carbohydrate-insulin model revealed that a high-refined carbohydrate diet can cause rapid spikes in blood sugar levels, causing elevated insulin secretion, which may promote fat deposition and inhibit fat breakdown, raising the risk of obesity [42]. Another possible explanation is the excessive consumption of poultry and red meat. Dietary pattern trajectory studies have found that diets high in red and processed meats may increase obesity risk [43]. Long-term high meat consumption patterns were also found to increase the risk of obesity [44]. Meat intake has been linked to an increase in specific metabolites in blood lipid levels, which may alter lipid metabolism pathways and positively correlate with obesity risk, promoting its development [45]. Although CRP level was positively correlated with the risk of childhood obesity, our study found no evidence of an indirect effect of CRP level on the association between rice and meat pattern and childhood obesity. The high intake of poultry may have obscured the effects of refined carbohydrates and red meat on CRP since some studies have found a positive correlation between refined carbohydrates and red meat intake and CRP levels in children [46], while poultry intake may have a negative correlation [47]. The complex effects of food combinations, such as refined rice, poultry, red meat, and meat products, demonstrate the advantage of analyzing dietary patterns rather than individual food groups. Another possibility is that rice and meat pattern may increase obesity risk in children through lipid metabolism pathways, rather than the CRP pathway [45]. CRP levels can also be influenced by various factors, including sedentary behavior and lack of physical activity.

The fruit and vegetable pattern was characterized by fresh fruits, vegetables, whole grains, fungi, and algae, reflecting the abundance of vegetables and fruit diversity typical of the Lingnan region in China. Other studies have found that plant-based or vegetarian dietary patterns had no significant total effect on general obesity, which is consistent with our findings [18]. Our research showed that this pattern had a significant indirect effect on reducing the obesity risk in children by lowering CRP levels. The high intake of fruits and vegetables, which has been linked to lower CRP levels in children, might explain this effect [46]. Extensive research has linked the Mediterranean diet, which is rich in fruits and vegetables to lower inflammatory levels and reduce the risk of obesity [48]. Fruits and vegetables are rich in vitamins, minerals, dietary fiber, and phytochemicals. These nutrients can reduce inflammation in the body by scavenging free radicals, inhibiting inflammatory enzymes, producing butyrate, and regulating the gut microbiota [49]. For example, flavonoids reduce pro-inflammatory factors such as IL-6, IL-1, and TNF-α by inhibiting the activation of NF-κB and NLRP3 inflammasomes, while also activating the AMPK pathway, thereby lowering CRP levels and exerting anti-inflammatory effects [50]. A population-based gut microbiome study found that a higher adherence to the Mediterranean diet is associated with lower CRP levels, a correlation that may be related to an increase in the abundance of the Porphyromonadaceae family and a decrease in the abundance of the Peptostreptococcaceae family [51]. The Mediterranean diet’s reduction in obesity risk is closely related to the anti-inflammatory effects of its rich polyphenols. This is mainly because these polyphenols can inhibit the activity of inflammatory factors such as TNF-α and NF-κB, reduce oxidative stress, and upregulate antioxidant and anti-inflammatory molecules, thereby alleviating chronic inflammation associated with obesity [52]. In addition, the Mediterranean diet reduces the accumulation of abdominal fat by modulating the gut microbiota, particularly by increasing the abundance of Porphyromonadaceae and decreasing that of Peptostreptococcaceae, promoting the production of short-chain fatty acids, enhancing the function of the intestinal barrier, and reducing inflammation [51]. Although the direct effect of the fruit and vegetable pattern on obesity is not significant, these biologically active components could have a major influence through particular pathways. This finding suggests that increasing the consumption of fruits and vegetables may indirectly decrease the risk of obesity through anti-inflammatory pathways.

The snack pattern was characterized by convenience foods, beverages, ice cream, and fast food and reflected the increasing trend of salty snacks and beverage consumption among Chinese children [53]. Our study found that the snack pattern had a significant indirect effect on the obesity risk of children by increasing CRP levels, but the total effect on the risk of childhood obesity was not significant. This may be due to the opposing directions of the direct effect and the indirect effect mediated by CRP, which could suppress the influence of the snack pattern on obesity. Inflammation is often considered to be a consequence of obesity. However, growing evidence suggests that there is a bidirectional relationship between inflammation and obesity [54]. This finding implies that prior inflammatory states can induce subsequent weight changes. Previous studies have linked the intake of high-salt and high-sugar foods to elevated levels of inflammatory markers, consistent with our findings [55]. Cohort studies have shown a positive correlation between an elevated dietary inflammatory index at 5 years of age and a greater risk of obesity at 9 years of age [56]. A multinational study among children and adolescents demonstrated that the consumption of ultra-processed foods could be a potential determinant of childhood obesity [57]. A diet high in sugar, fat, and trans fats exacerbates oxidative stress, triggers the production of inflammatory mediators, disrupts the homeostasis of the gut microbiota, impairs the functionality of the intestinal barrier, and reshapes the activity of immune cells, collectively driving the exacerbation of systemic inflammatory responses [58]. Evidence suggests that a high-sugar diet promotes Th17 cell differentiation by activating the TGF-beta and NF-kB signaling pathways, disrupts the balance of the gut microbiota, reduces short-chain fatty acids, and thereby increases intestinal permeability, leading to inflammation [59]. Trans fat intake activates Toll-like receptor 4 (TLR4) and the downstream NF-κB signaling pathway, increases lipopolysaccharide (LPS) levels in the serum, activates the TLR4-CD14 system, and promotes the release of inflammatory cytokines, leading to inflammation [60]. A study involving the genetic ablation of CRP in rats has shown that CRP is not only a biomarker for metabolism and inflammation but also directly affects energy balance, body weight, insulin sensitivity, and glucose homeostasis by regulating central leptin effects and hypothalamic signaling, thereby influencing the development of obesity [61]. Existing studies show that pro-inflammatory diets increase Gram-negative bacteria in the gut, activating Toll-like receptors, elevating inflammation levels, inducing endoplasmic reticulum stress in adipocytes to activate the UPR, JNK, and NF-κB pathways, and promoting fat accumulation, thereby linking to obesity [16]. Our study also discovered a significant direct effect of the snack pattern on lowering obesity risk in children, which might be explained by their high beverage intake. Surveys have shown that the consumption of dairy beverages ranks second among Chinese children [62]. Our previous research has revealed that dairy-containing patterns might increase the risk of malnutrition in children, since dairy beverages may suppress appetite, lower the consumption of core foods, and result in insufficient total energy intake [63]. The epidemiological data on the association between snacks and obesity risk has been inconsistent [64]. Our study suggests that increased snack intake may indirectly increase obesity risk through pro-inflammatory pathways.

The strength of this study is its use of mediation analysis to identify the potential role of CRP in the association between dietary patterns and obesity. However, our study had some limitations. First, CRP only contributes to a part of the association between diet and obesity, and further research is needed to investigate other potential mediators. Second, the study sample of children aged 9–17 years from rural Guangzhou may not be representative of all Chinese children. Given the potential disparities between populations, these findings should be inferred with caution. Third, using FFQs to gather dietary data may introduce a recall bias. Given the potential bias of self-reported dietary and physical activity data, future nutrition studies should integrate objective methods such as metabolomics to enhance the accuracy and reliability of the findings. Fourth, a cross-sectional study could not establish causality. Finally, despite adjusting for several potential sociodemographic and behavioral confounders in our multivariate models, residual confounding may still exist because of unmeasured or insufficiently measured factors.

## 5. Conclusions

In summary, after adjusting for confounding factors, the rice and meat pattern was significantly associated with the risk of childhood obesity. Our study offers cross-sectional evidence for the role of CRP in the association between dietary patterns and childhood obesity risk. Future longitudinal and interventional studies are essential to further explore the causal relationship between dietary patterns and childhood obesity, with a particular emphasis on the role of inflammatory markers. In the daily lives of children and adolescents, it is necessary to ensure the intake of vegetables, fruits, and whole grains, while reducing the intake of high-fat, high-sugar, and high-salt foods to reduce the risk of obesity and inflammation.

## Figures and Tables

**Table 1:** Demographic and lifestyle characteristics of the study participants.

			General Obesity			Central Obesity		
	Characteristic	Total	No	Yes	*p*	No	Yes	*p*
Gender, n (%)					**0.046**			**0.016**
	girl	1116	1052 (94.3)	64 (5.7)		1020 (91.4)	96 (8.6)	
	boy	1297	1196 (92.2)	101 (7.8)		1147 (88.4)	150 (11.6)	
Age, medians (P_25_, P_75_) (year)		13.3 (11.3, 14.4)	13.3 (11.4, 14.4)	13.2 (10.9, 14.2)	0.212	13.2 (11.3, 14.4)	13.4 (11.2, 14.6)	0.362
Age group, n (%) (year)					0.138			0.521
	9–10	483	441 (91.3)	42 (8.7)		431 (89.2)	52 (10.8)	
	11–13	1122	1050 (93.6)	72 (6.4)		1020 (90.9)	102 (9.1)	
	14–17	808	757 (93.7)	51 (6.3)		716 (88.6)	92 (11.4)	
School stage, n (%)					0.678			0.549
	primary school	994	921 (92.7)	73 (7.3)		897 (90.2)	97 (9.8)	
	junior high school	1044	975 (93.4)	69 (6.6)		939 (89.9)	105 (10.1)	
	senior high school	375	352 (93.9)	23 (6.1)		331 (88.3)	44 (11.7)	
Lifestyle, n (%)					0.986			0.967
	Health group	1196	1115 (93.2)	81 (6.8)		1076 (90.0)	120 (10.0)	
	Poor sleep group	1053	980 (93.1)	73 (6.9)		944 (89.6)	109 (10.4)	
	Risk group	164	153 (93.3)	11 (6.7)		147 (89.6)	17 (10.4)	
Breakfast, n (%) (time/week)					**<0.001**			**<0.001**
	≤3	180	154 (85.6)	26 (14.4)		147 (81.7)	33 (18.3)	
	>3	2233	2094 (93.8)	139 (6.2)		2020 (90.5)	213 (9.5)	
Education of father, n (%)					0.269			0.219
	Junior high school or below	1132	1060 (93.6)	72 (6.4)		1021 (90.2)	111 (9.8)	
	High school	706	658 (93.2)	48 (6.8)		640 (90.7)	66 (9.3)	
	College degree or above	575	530 (92.2)	45 (7.8)		506 (88.0)	69 (12.0)	
Education of mother, n (%)					**0.047**			0.086
	Junior high school or below	1239	1165 (94.0)	74 (6.0)		1125 (90.8)	114 (9.2)	
	High school	584	543 (93.0)	41 (7.0)		521 (89.2)	63 (10.8)	
	College degree or above	590	540 (91.5)	50 (8.5)		521 (88.3)	69 (11.7)	
Nature of school, n (%)					**0.001**			**0.021**
	Public school	2052	1926 (93.9)	126 (6.1)		1855 (90.4)	197 (9.6)	
	Private school	361	322 (89.2)	39 (10.8)		312 (86.4)	49 (13.6)	
Body mass index, medians (P_25_, P_75_) (kg/m^2^)		18.2 (16.4, 20.5)	18.0 (16.3, 19.9)	27.1 (24.9, 29.1)	**<0.001**	17.9 (16.2, 19.8)	25.0 (23.2, 28.2)	**<0.001**
Waist circumference, medians (P_25_, P_75_) (cm)		62.5 (58.0, 68.0)	62.0 (57.5, 66.8)	81.8 (75.8, 89.6)	**<0.001**	61.5 (57.4, 66.0)	81.3 (76.5, 88.0)	**<0.001**

Note: Data are presented as numbers (percentage) for categorical variables and as medians (P_25_, P_75_) for continuous variables. Intergroup differences were compared using chi-square tests and Mann–Whitney U tests. Bold *p*-values indicate “<0.05”.

**Table 2:** Analysis of association between dietary patterns, CRP, and general and central obesity.

Dietary Pattern/LnCRP	General Obesity						Central Obesity					
	Model 1			Model 2			Model 1			Model 2		
	OR	95%CI	*p*	OR	95%CI	*p*	OR	95%CI	*p*	OR	95%CI	*p*
Fruit and vegetable pattern	1.103	0.956, 1.272	0.180	1.096	0.947, 1.269	0.220	0.972	0.848, 1.114	0.684	0.961	0.837, 1.103	0.574
Snack pattern	0.916	0.773, 1.085	0.309	0.879	0.738, 1.046	0.145	0.907	0.785, 1.047	0.181	0.884	0.764, 1.024	0.100
Rice and meat pattern	1.138	0.976, 1.327	0.098	1.166	1.000, 1.359	**0.049**	1.197	1.056, 1.357	**0.005**	1.215	1.071, 1.377	**0.002**
LnCRP	2.167	1.683, 2.789	**<0.001**	2.301	1.776, 2.982	**<0.001**	2.104	1.696, 2.611	**<0.001**	2.165	1.738, 2.697	**<0.001**

Note: OR stands for odds ratio, and 95% CI stands for 95% confidence interval. Bold *p*-values indicate “<0.05”. Model 1 is adjusted for gender and age, while Model 2 is adjusted for lifestyle, frequency of breakfast consumption, and type of school attended based on Model 1.

**Table 3:** Analysis of association between dietary patterns and CRP.

Dietary Pattern	Model 1			Model 2		
	β	95%CI	*p*	β	95%CI	*p*
Fruit and vegetable pattern	−0.064	−0.086, −0.041	**<0.001**	−0.059	−0.081, −0.036	**<0.001**
Snack pattern	0.051	0.028, 0.073	**<0.001**	0.043	0.020, 0.065	**<0.001**
Rice and meat pattern	0.031	0.007, 0.056	**0.011**	0.024	0.000, 0.048	0.055

Note: β represents the unstandardized regression coefficient, and 95% CI denotes the 95% confidence interval. Bold *p*-values indicate “<0.05”. Model 1 is adjusted for gender and age, whereas Model 2 is adjusted for lifestyle, frequency of breakfast consumption, and the nature of the school attended based on Model 1.

**Table 4:** Mediation analysis of CRP on the relationship between dietary patterns and general obesity.

Dietary Pattern	Total Effect			Indirect Effect			Direct Effect			Conclusion
	β	95%CI	*p*	β	95%CI	*p*	β	95%CI	*p*	
Fruit and vegetable pattern	0.00603	−0.00418, 0.02	0.25	−0.00306	−0.00462, 0.00	**<0.001**	0.00909	−0.00129, 0.02	0.09	Suppression
Snack pattern	−0.00720	−0.01821, 0.00	0.149	0.00270	0.00131, 0.00	**<0.001**	−0.00991	−0.02075, 0.00	**0.043**	Suppression
Rice and meat pattern	0.0109	0.0000627, 0.02	**0.037**	0.001180	−0.0000279, 0.00	0.054	-	-	-	-

Note: β denotes the unstandardized regression coefficient, and 95% CI refers to the 95% confidence interval. Bold *p*-values indicate “<0.05”. A dashed line indicates that the indirect effect is insignificant, resulting in the termination of the mediation analysis. Adjusted variables include gender, age, lifestyle, frequency of breakfast consumption, and the nature of the school attended.

**Table 5:** Mediation analysis of CRP on the relationship between dietary patterns and central obesity.

Dietary Pattern	Total Effect			Indirect Effect			Direct Effect			Conclusion
	β	95%CI	*p*	β	95%CI	*p*	β	95%CI	*p*	
Fruit and vegetable pattern	−0.00354	−0.016925, 0.01	0.58	−0.003797	−0.005659, 0.00	**<0.001**	0.000256	−0.01325, 0.01	0.98	Suppression
Snack pattern	−0.00937	−0.02410, 0.00	0.138	0.00363	0.00185, 0.01	**<0.001**	−0.01300	−0.02744, 0.00	**0.034**	Suppression
Rice and meat pattern	0.0196	0.00694, 0.03	**0.002**	0.00157	−0.000069, 0.00	0.061	-	-	-	-

Note: β denotes the unstandardized regression coefficient, and 95% CI refers to the 95% confidence interval. Bold *p*-values indicate “<0.05”. A dashed line indicates that the indirect effect is insignificant, resulting in the termination of the mediation analysis. Adjusted variables include gender, age, lifestyle, frequency of breakfast consumption, and the nature of the school attended.

## Data Availability

Due to privacy concerns, the data used in this study are not publicly available. The data can be obtained by contacting the corresponding author.

## Supplementary Materials

The following supporting information can be downloaded at: https://www.mdpi.com/article/10.3390/nu16223835/s1, Table S1: Food groups used in the factor analysis; Table S2: The variables and their assigned values included in the lifestyle model; Table S3: Fit indices of the latent class model for lifestyle; Table S4: Factor analysis yields the factor loadings of the 20 food groups and the dietary patterns. Figure S1: Scree plot.
