# Supplementary material for: Association of Dietary Patterns, C-Reactive Protein, and Risk of Obesity Among Children Aged 9–17 Years in Guangzhou, China: A Cross-Sectional Mediation Study
Source: Nutrients. 2024 Nov 8;16(22):3835. doi: 10.3390/nu16223835 (PMC11597664; doi:10.3390/nu16223835)
Supplement: Supplementary file 1 [file nutrients-16-03835-s001.zip › nutrients-3261591-supplementary.pdf]

**TITLE: Association of dietary patterns, C-reactive protein, and risk of obesity among children aged 9-17 years in Guangzhou, China: A Cross-Sectional Mediation Study**

**SUPPLEMENTARY MATERIALS**

**Table S1: Food groups used in the factor analysis**

| Number | Food Group | Examples of Food Items |
|---|---|---|
| 1 | Rice and rice products | Rice, rice gruel, rice noodles |
| 2 | Wheat and wheat products | Wheat flour noodles, wheat buns, dumplings Fried bread, fried breadsticks |
| 3 | Coarse | Corn, cornmeal, potato, taro, sweet potato |
| 4 | Beans and bean products | Soybean, soybean milk, tofu, bean curd, dried bean curd |
| 5 | Fresh vegetables | Turnip, lettuce, cauliflower, cabbage, Snow peas |
| 6 | Mushrooms and algae | Mushroom, laver, kelp |
| 7 | Fresh fruits | Orange, pear, pineapple, watermelon |
| 8 | Mike and dairy products | Milk, milk powder, yogurt, cheese |
| 9 | Red meat and meat products | Pork, beef, goat, lamb, ham sausage, bacon, meatball |
| 10 | Poultry | Chicken, duck, goose |
| 11 | Animal organ | Liver, heart, kidney, lung, intestine |
| 12 | Aquatic products | Fish, shrimp, crab |
| 13 | Eggs | Eggs |
| 14 | Nuts | Peanuts, chestnut, almonds, walnuts, hazelnuts |
| 15 | Baked goods | Cookies, cakes, bread, peach crisp |
| 16 | Candy | Sugar, jam, jelly, candies, chocolate, candied fruit |
| 17 | Fast food | Hamburger, fried chicken |
| 18 | Snack convenience food | Instant noodles, instant rice noodles, Spicy strips, fried puffed snacks |
| 19 | Beverages | Carbonated drinks, prepackaged juice, milk beverages, sweet tea beverages, sports beverages, zero-sugar drinks |
| 20 | Ice cream | Ice cream, cone, sundae |

**Table S2: The variables and their assigned values included in the lifestyle model**

| Number | Variable | Assign a value of 0 | Assign a value of 1 |
|---|---|---|---|
| 1 | Tried smoking | No | Yes |
| 2 | Alcohol consumption | No | Yes |
| 3 | Moderate-to-high physical activity | ＜3day/week | ≥3 day/week |
| 4 | Screen time | ＜2hour/day | ≥2 hour/day |
| 5 | Sleep time | Insufficient | Sufficient |
| 6 | Bed time | ＜22：30 | ≥22：30 |

**Table S3: Fit indices of the latent class model for lifestyle**

| Model | AIC | BIC | ABIC | Entropy | LMR (P-value) | BLRT (P-value) | Mixing ratio |
|---|---|---|---|---|---|---|---|
| M2 | 15782.655 | 15857.908 | 15816.604 | 0.495 | < 0.001 | < 0.001 | 0.444/0.556 |
| M3 | 15622.144 | 15737.917 | 15674.372 | 0.684 | < 0.001 | < 0.001 | 0.496/0.436/0.068 |
| M4 | 15614.447 | 15770.740 | 15684.954 | 0.537 | 0.7847 | 0.0300 | 0.233/0.047/0.237/0.483 |
| M5 | 15614.476 | 15811.289 | 15703.263 | 0.666 | 0.0116 | 0.1304 | 0.042/0.239/0.078/0.123/0.518 |

Note: The analysis employed latent class analysis. AIC refers to the Akaike Information Criterion, BIC refers to the Bayesian Information Criterion, ABIC refers to the Adjusted Bayesian Information Criterion, LMR refers to the Lo-Mendell-Rubin Adjusted Likelihood Ratio Test, BLRT refers to the Bootstrap Likelihood Ratio Test, Entropy refers to the average amount of information, and Mixing ratio refers to the proportion of each category.

**Table S4: Factor analysis yields the factor loadings of the 20 food groups and the dietary patterns**

| Food Group | Fruit and vegetable pattern | Snack pattern | Rice and meat pattern |
|---|---|---|---|
| Fresh fruits | 0.685 | 0.168 | 0.113 |
| Fresh vegetables | 0.669 | 0.021 | 0.284 |
| Coarse | 0.626 | 0.022 | 0.034 |
| Mushrooms and algae | 0.566 | 0.143 | 0.092 |
| Beans and bean products | 0.474 | 0.118 | 0.306 |
| Aquatic products | 0.386 | 0.300 | 0.251 |
| Nuts | 0.373 | 0.304 | -0.040 |
| Snack convenience food | 0.010 | 0.696 | 0.133 |
| Beverages | 0.203 | 0.695 | 0.181 |
| Ice cream | 0.005 | 0.568 | -0.041 |
| Fast food | 0.043 | 0.562 | 0.194 |
| Candy | 0.257 | 0.550 | -0.072 |
| Baked goods | 0.113 | 0.362 | 0.070 |
| Mike and dairy products | 0.174 | 0.310 | 0.224 |
| Rice and rice products | -0.105 | 0.074 | 0.662 |
| Poultry | 0.107 | 0.054 | 0.560 |
| Red meat and meat products | 0.229 | 0.189 | 0.521 |
| Animal organ | 0.097 | -0.060 | 0.476 |
| Eggs | 0.196 | 0.189 | 0.408 |
| Wheat and wheat products | 0.263 | 0.125 | 0.334 |

Note: The analysis employed Principal Component Analysis.

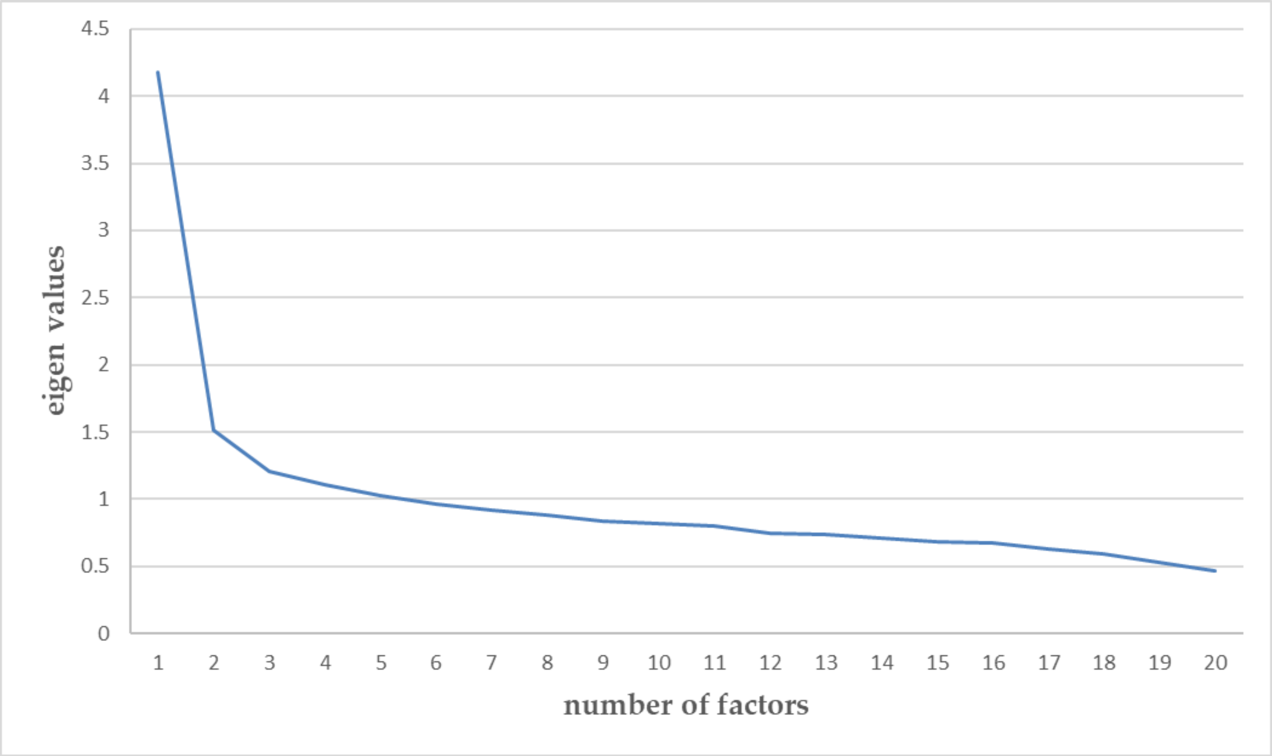


**Figure S1: Scree plot**
